# Interfacial Water as a Chemically Tunable Medium: From Molecular Structure to Materials Function

**DOI:** 10.3390/molecules31213851

**Published:** 2026-10-26

**Authors:** Ulzhalgas Nazarbek, Yerkebulan Raiymbekov, Saule Nazarbekova, Perizat Abdurazova, Galiya Kambarova

**Affiliations:** 1 U. Besterekov “EcoGreenLab”, M. Auezov South Kazakhstan University, 160012 Shymkent, Kazakhstan; unazarbek01@gmail.com (U.N.); nazarbek.s.p@mail.ru (S.N.); galiakambarova8@gmail.com (G.K.); 2 Department of Chemistry, Zhanibekov University, 160012 Shymkent, Kazakhstan; abdurazova.perizat@okmpu.kz

**Keywords:** interfacial water, hydration, ion-specific effects, Hofmeister series, nanoconfinement, electrical double layer

## Abstract

Modern instruments used to observe water at interfaces can distinguish individual molecular layers, measure dielectric permittivity within nanometer-scale gaps, and track reorientation on femtosecond timescales. Yet, a materials scientist asking whether increasing the density of ionizable groups would improve membrane performance finds little direct guidance in this literature. We attribute this disconnect to three factors: the term “structure” refers to three independent properties of water; the parameters typically controlled are not actually independent variables; and the state of water influences material performance through multiple pathways that yield opposing effects. We synthesize the accumulated evidence regarding the interdependence of five factors, show how ion-series reversal can serve as a screening indicator of conditional coupling when alternative mechanistic causes are excluded, and explain why a specific change in the state of water can enhance performance in some areas while impairing it in others. This framework has implications for the design of membranes, sorbents, and coatings and suggests that many of the most pressing challenges in the field can be addressed using existing tools, provided that greater emphasis is placed on how measurements are reported and experiments are designed rather than solely on acquiring new data.

## 1. Introduction

Observing interfacial water has become a precise endeavor. The distribution of molecules near a solid surface is mapped in three dimensions with molecular resolution [1], dielectric permittivity is measured in nanometer-wide gaps [2], the reorientation of individual groups is tracked on femtosecond timescales [3], and an ion’s hydration number can be directly linked to its mobility along the surface [4].

Yet, the existing literature fails to answer the questions a materials scientist actually asks: Will increasing the density of ionizable groups improve membrane performance? Is it worth aiming for stronger water binding within a sorbent? Can a solution found for one polymer be applied to another? The gap between the ability to characterize a system and the ability to prescribe a course of action does not narrow as instrument resolution improves; it is the nature of this gap that we examine here.

This difficulty is most clearly illustrated by a frequently encountered proposition: that “more ordered” interfacial water enhances material function. Here, the quotation marks are deliberate, because such wording is not operationally meaningful unless “order” is tied to a specific observable, such as orientational alignment, hydrogen-bond strength, or molecular dynamics. This proposition is supported in some contexts but contradicted in others, with the various cases failing to form a consistent pattern. Such discrepancies are usually attributed to differences between the systems involved. While correct, this explanation is ultimately unproductive, as it implies that generalization is impossible.

Three recurring problems motivate the organization of this review: ambiguity in what is meant by water “structure”, coupling between variables that are often treated as independent, and the existence of multiple pathways linking water state to material function. These issues are addressed in Section 2, Section 3, Section 4, Section 5, Section 6 and Section 7.

This review addresses the question of which molecular factors determine the state of interfacial water and how that state translates into material function. These five factors define the principal chemical variables through which the state of interfacial water can be tuned. Their effects, however, are coupled rather than independent, so changing one factor may simultaneously shift the system along one or more of the others.

Previous reviews have established several of the individual elements considered here. Gonella et al. [5] provided a molecular-level synthesis of water at charged metal, oxide, and biomembrane interfaces and emphasized the limitations of classical mean-field descriptions. We take this work as a starting point but address a different question. The present review does not propose surface chemistry, charge, ion identity, confinement, or the thermodynamic environment as new determinants. Its distinctive contribution is to organize these established determinants as a coupled, non-orthogonal parameter space, to make their conditional dependencies explicit, and to connect them to structure–dynamics relationships and material function. Related reviews focused on surface hydration, ion-specific effects, and confined water are compared with this framework in the corresponding sections below.

The scope of this review is deliberately selective. It is a narrative, hypothesis-oriented synthesis rather than a comprehensive or systematic survey of interfacial-water research. We focus on studies relevant to surface chemistry, surface charge, ion identity, spatial confinement, and the thermodynamic or chemical environment, particularly where these factors can be related to water structure, dynamics, transport, or material function. Representative systems include mineral and oxide interfaces, functionalized polymers, membranes, nanopores, sorbents, electrode interfaces, and protective or antifouling coatings. The discussion is focused primarily on equilibrium and steady-state conditions, while methodological studies are included only when they affect the interpretation, comparability, or design relevance of interfacial-water measurements.

The literature was identified through bibliographic searches covering surface hydration, ion-specific effects, Hofmeister behavior, water under confinement, polymer–water interfaces, and related combinations of these terms. DOI records were used to verify bibliographic information and consolidate duplicate records. Studies were selected for detailed discussion when they met at least one of three criteria: they provided direct evidence for a proposed relationship, defined a limit or boundary condition for that relationship, or documented an apparent contradiction useful for distinguishing competing interpretations. This approach intentionally favors mechanistically informative studies and should therefore not be interpreted as a statistically representative sampling of the entire field. Claims concerning prevalence or frequency are restricted to the literature surveyed here unless supported by an explicit quantitative analysis.

This selection strategy has an important limitation: the resulting literature sample is not statistically representative of the field. Because preference was given to studies that discriminate between competing interpretations, establish limits of applicability, or reveal apparent contradictions, reports of reversals, decoupling, and other mechanistically informative outcomes may be overrepresented relative to confirmatory or null-result studies. Consequently, statements concerning how frequently particular practices, mechanisms, or evidence gaps occur should be interpreted as qualitative observations within the literature surveyed here unless an explicit count is provided. The framework is therefore intended to organize mechanistic evidence and generate testable questions, rather than to estimate the prevalence of specific behaviors across the entire literature.

## 2. What Is Meant by “Interfacial Water”

### 2.1. The Source of the Terminological Confusion

The field operates using a set of terms that are almost never defined. Researchers speak of structured, ordered, bound, non-freezing, vicinal, and interfacial water, as well as hydration shells and exclusion zones; in many studies, two or three of these expressions are used interchangeably. It would be incorrect to attribute this simply to carelessness.

The confusion stems from methodological origins. Each term emerged within a specific experimental tradition, where it held a precise meaning defined by the measurement procedure. “Bound water” referred to the fraction that did not exhibit a thermal effect of melting during calorimetric experiments. “Ordered water” implied a preferential orientation detected via a nonlinear optical response. A “hydration shell” denoted the set of molecules within an ion’s coordination spheres. Upon leaving the tradition that spawned it, a term would lose its link to the specific procedure while retaining only a general qualitative meaning; consequently, the terms became interchangeable, and the underlying physical quantities ceased to be distinguished.

This is not merely a matter of precise language. The assertion that water near a given surface is “more structured” lacks verifiable content and thus can be neither confirmed nor refuted; furthermore, a significant portion of the discrepancies discussed in Section 6 arises because distinct physical quantities are designated by the same term.

### 2.2. Terms and Their Operational Content

A summary of commonly used terms, indicating the tradition from which they originate, is provided below (Table 1). The fourth column indicates what the term does not mean, as the unjustified expansion of meaning is a typical source of error.

These terms do not form a hierarchy, nor can they be reduced to one another. “Bound water” is defined by an exchange-time threshold or a thermal effect; “ordered water” by response symmetry; and the “hydration shell” by coordination. The quantities underlying these definitions vary independently, and the convergence of conclusions derived from different definitions would require as much explanation as their divergence.

### 2.3. Three Independent Properties Conflated by the Word “Structure”

The word “structure” causes the most confusion, as it masks at least three logically independent properties. Orientational order refers to the presence of a preferred direction for the dipole moment or OH groups relative to the surface normal. Hydrogen-bond strength is gauged by shifts in vibrational frequency. Dynamic sluggishness is inferred from relaxation times and mobility coefficients. There is no necessary link between them; thus, a statement about “more structured water”—without specifying which property is meant—allows for three mutually incompatible interpretations.

This independence is confirmed by direct observations, discussed in detail in Section 4. Suffice it to say that a charged surface enhances the first property—with the ordering reaching a plateau above a certain charge-density threshold—whereas bond strength and mobility continue to change; furthermore, in films of carboxylated cellulose nanofibrils, a nearly threefold change in charged-group density leaves water dynamics unchanged [6].

Atomically resolved studies of calcite provide an example of strongly ordered hydration layers: simulations and force microscopy show distinct molecular arrangements in successive interfacial layers [7,8]. These studies demonstrate structural ordering at the molecular scale but do not, by themselves, establish the magnitude of a nonlinear optical response. They are therefore used here only as an example of spatially resolved interfacial order.

The practical conclusion is simple. The word “structure” should be replaced by a specification of the particular property and the quantity used to measure it. While this slightly increases the length of the text, it renders statements verifiable and eliminates a significant number of apparent discrepancies.

### 2.4. Where Interfacial Water Ends

The question of the extent of the region to be classified as interfacial water is usually posed as if it had a single answer. Estimates in the literature vary by several orders of magnitude, a fact often taken as evidence of the subject’s inherent ambiguity. This variation, however, is natural, since different properties decay over different distances, and the extent is defined only in conjunction with the specific property and the decay criterion (Table 2).

Water density oscillations along the normal to a mica interface have been established via X-ray reflectometry, with the limiting oxygen atom density matching the bulk value—a feature that defines the interface boundary [9]; layering is also observed in biomembranes [10] and can be resolved in three dimensions using force microscopy [1]. The hydration force between approaching surfaces decays over the span of a few molecular layers [11]. Various estimates are consistent, indicating a range on the order of the first few layers [12], while direct measurements at an orthoclase interface demonstrate that solution composition alters both the near-surface liquid structure and the magnitude of these forces [13].

Orientational ordering induced by a charged surface behaves differently, as it is governed by the extent of the interfacial field—specifically, the Debye length. This length varies from the nanometer scale at physiological ionic strength to the micrometer scale for a charged mineral at near-neutral pH and very low salt concentration [5]. This three-order-of-magnitude difference does not represent a contradiction; rather, it reflects the same property under varying conditions, and the dependence of this range on ionic strength is a subject of study in itself, not a complication. A dielectric anomaly is observed at the nanometer scale [2], and deviations in mobility from bulk values are typically confined to the first few layers, though this depends on the specific system (Figure 1). There is no single, universal thickness for interfacial water; citing a value without specifying the property in question is meaningless.

## 3. Five Factors of Interfacial Water and a Map of Their Interdependencies

### 3.1. Five Factors and the Connections Between Them

Discussions of interfacial water are traditionally structured around a list of influencing factors, treating surface hydrophilicity, surface charge, the nature of dissolved ions, geometric confinement, and thermodynamic conditions as separate entities. While this organization reflects the actual division of research communities, it carries the implicit assumption that these quantities are independent variables whose contributions are additive.

We structure this section differently. The five quantities—referred to here as “factors”—define the space in which the state of interfacial water exists. However, the focus of this section is not the factors themselves—the list of which has long been known in various formulations—but the “edges” connecting them. Accordingly, the contribution of the present framework is not a new taxonomy of determinants, but an explicit representation of their experimentally supported conditional dependencies. We define an “edge” as an established dependency wherein a change in one factor automatically alters the system’s position regarding another, making it impossible to vary them independently in an experiment without taking special measures. The set of these edges forms a map of interdependencies; constructing this map, along with the method for identifying such interdependencies based on observed behavior, constitutes the content of this section (Figure 2).

The axes are defined as follows. The first describes surface chemistry—specifically, the type and density of functional groups, their hydrogen-bonding donor–acceptor capability, and local polarity. The second describes surface charge and the resulting interfacial electric field. The third describes the nature of the ions present, including charge density, hydration number, and dehydration energetics. The fourth describes spatial confinement—that is, the characteristic size of a cavity or gap relative to the molecular scale of water. The fifth describes the thermodynamic and chemical environment, encompassing temperature, ionic strength, water activity, pH, and the presence of co-solvents.

### 3.2. Established Foundation

Two foundational couplings can be considered firmly established; detailing them fully would require repeating material already summarized in the literature [5].

The first pillar links surface chemistry to surface charge. The charge on oxides and functionalized polymers arises from the acid–base equilibrium of specific groups; silanols simultaneously determine the acidity of silica and modulate the properties of adjacent water [14], while the surface pKa of quartz depends on the structure of the interface [15]. The water response is asymmetric with respect to the sign of the charge: introducing a positive charge into zwitterionic monolayers disrupts water structure, whereas a negative charge reinforces it [16]—meaning that this factor cannot be described by a single scalar quantity. The relationship between surface charge and hydration on mineral surfaces has been critically reviewed elsewhere [17]. That work focused on the charge–hydration relationship within mineral systems, whereas in the present framework, this relationship represents one coupling edge within a broader network that also includes ion identity, confinement, and the thermodynamic environment. The orientational behavior of water can be traced directly to surface chemistry [18] and remains sensitive even during crystallization [19]; for aluminum hydroxides, hydration is determined by the configuration of hydroxyl groups on the crystal face rather than the bulk composition [20], while on charged monolayers, electrostatics, hydration, and chemical equilibrium are coupled into a unified response [21].

The second pillar links surface charge to the nature of the ion. Ion adsorption can not only screen the charge but also reverse its sign—a phenomenon established over half a century ago regarding the charge reversal of oxide colloids by hydrolyzable cations [22] and subsequently observed at the molecular level at the silica–divalent electrolyte solution interface [23]. A more subtle aspect of this phenomenon is that the counterion alters the acid–base state of the surface itself, as the relative populations of silanol groups depend on the specific cation present [24], while halide anions induce a cooperative change in the acid–base response [25]. Charge transfer between the ion and the surface gives rise to ion-specific effects even at low salt concentrations [26].

### 3.3. The Medium Redefines the Ion Order

The link between the nature of the ion and the nature of the medium provides the clearest evidence of coupling, as it is the *order* of the effect—rather than its magnitude—that changes here. Sum-frequency generation spectroscopy at the silica/alkali metal chloride solution interface (at a salt concentration of 0.5 mol/L) revealed a direct cation adsorption order (from lithium to cesium) at pH 8, a reversal of this order above pH 10, and a second trend shift below pH 6—the latter associated with cation hydration asymmetry and double-layer charge reversal [27]. The surface and the set of ions remain constant throughout; only the position along the medium-related factor changes.

Other environmental elements achieve the same result. Osmolytes regulate the effective surface charge and, through it, ion specificity [28,29]; a change in solvent yields a reversed series at the formamide interface [30]; an applied field alters ionic effects on mineral colloid aggregation [31]; and flow along the surface reversibly modifies its interfacial chemistry [32]. The nature of the cation critically influences the state of water at the negatively charged silica surface, an effect that cannot be reduced merely to charge magnitude [33]. Ion-specific and Hofmeister effects have been reviewed extensively in protein and lipid model systems [34,35]. These reviews establish the breadth and molecular origins of ion-specific behavior, whereas the present framework treats ion identity as one coupled factor whose effect may change with surface state, medium, or confinement. The emphasis here is therefore not on defining a universal ion sequence, but on identifying when and why ion ranking becomes conditional on another factor. An ion’s action depends on other solution constituents—even buffer components, which make their own contributions [36]. Colloid aggregation series reproduce the Hofmeister sequence only partially [37], and anions ranging from chloride to thiocyanate yield distinctly different patterns at charged interfaces [38]. Specificity is mediated by water rather than direct contact [39]; water-mediated attraction orders adsorbed alkali ions [40], and competitive adsorption gives rise to a salt effect that cannot be reduced to individual contributions [41]. Ion effects are also evident in the collective dynamics of water [42,43], while the coexistence of kosmotropic and chaotropic urea effects within a single system [44] highlights the inadequacy of a one-dimensional scale. However, the molecular underpinnings of specificity—anion polarizability [45], many-body ion–water interactions [46], the absolute hydration energy scale [47], and non-classical cation polarization [48]—have been less thoroughly investigated than the phenomenology; in soft matter systems, these same differences manifest as the dependence of brush hydration on the counter-anion [49], the restoration of salt-screened interactions by osmolytes [50], and the impact of divalent cations on lipid interfaces [51].

### 3.4. Confinement as an Independent Factor Rather than Merely an Experimental Geometry

Spatial confinement is most often treated as a calculation condition—specifically, a fixed distance between two walls within a molecular dynamics cell. In this formulation, confinement acts as a model parameter rather than a governing factor, and the question of its coupling with other factors is not raised. Accumulated data, however, indicate that confinement alters the very medium in which other factors operate.

This is most directly evident in the dielectric response. Local capacitance measurements of water confined between atomically flat walls revealed an anomalously low out-of-plane dielectric component, *ε*_⊥_, in the interfacial region [2]. This result should not be interpreted as a single scalar dielectric constant for confined water: dielectric response under confinement is anisotropic and depends on field orientation and geometry. Confinement can therefore modify electrostatic interactions, but the magnitude and direction of this modification are geometry- and component-dependent.

A second line of evidence concerns ion distribution. Nuclear magnetic resonance studies of nanoconfined aqueous electrolytes have revealed a breakdown of local electroneutrality and associated ion-specific effects [52]; that is, confinement alters ion distribution in a manner that cannot be reduced to a simple reduction in available water volume or described merely by a renormalization of concentration. Related to this is the behavior of the screening length, which, contrary to classical expectations, increases with concentration in concentrated electrolytes [53]. Thus, results obtained for dilute solutions at extended surfaces should not be extrapolated directly to multi-molar electrolytes in nanopores without accounting for changes in dielectric response, screening, and ion distribution. Moreover, the screening length itself ceases to be a reliable guide in soft electrolyte systems [54], and ion profiles near dielectric inhomogeneities require accounting for non-electrostatic ion–surface interactions [55]. These observations should not be interpreted as quantitatively equivalent manifestations of confinement. They arise from systems spanning rigid nanogaps, soft electrolyte environments, biological nanopores, and chemically functionalized channels, with substantially different electrolyte concentrations, geometries, and measured quantities. Collectively, they support the conclusion that confinement modifies the operation of other factors, but they do not establish a single universal confinement law applicable across all interfaces.

A third line of evidence concerns transport and selectivity. In biological nanopores, the specific adsorption of trivalent cations determines conductance dynamics and selectivity reversal [56]; that is, the nature of the ion within the confined geometry governs the sign—rather than the magnitude—of the transport characteristic. In charged nanopores, selectivity depends on the combined action of several physical fields [57], making the separation of individual contributions non-trivial, even in a model setting.

Wall chemistry remains an independent factor even under confinement, with the governing parameter being not only the type of end group but also its surface coverage density. In nanofluidic channels based on transition metal carbides, a progressive increase in fluorine group coverage suppresses the transport of both cations and anions [58]. Functionalization of graphene interfaces controls the structure and dynamics of the confined electrolyte [59], while in multilayer membrane systems, functional groups simultaneously determine water flux and ion rejection [60]. Hydration cycles and wall polarity give rise to wetting hysteresis in silica nanochannels [61], adding a dependence on history to the overall picture.

Studies have also emerged that explicitly distinguish between interfacial contributions and the effects of nanoconfinement in charged nanopores [62], anisotropic dielectric responses [63], and field-driven dynamic permeability [64]; cavity geometry also governs water dynamics in lipid mesophases [65]. A recent review treats confined water as a distinct catalytic medium and emphasizes how confinement modifies thermodynamic properties and reaction kinetics [66]. The present framework assigns confinement a different role: it is one factor within a coupled parameter space, and the central question is how confinement modifies the effects of surface chemistry, charge, ion identity, and the surrounding environment rather than how confinement acts in isolation. In specific systems, the link between these factors and confinement is clearly evident: the interaction of monovalent ions with graphene oxide is governed by carboxyl groups rather than the sheet’s overall charge [67]; transport in calcium silicate nanopores is tuned by wall chemistry [68]; and in conically shaped nanopores with bipolar coatings, transport depends on the location of the junction between segments [69]. Metal-organic frameworks can distinguish between ions of the same charge at sub-nanometer scales [70]; in transition-metal carbide channels, selectivity is mediated by water [71]; and transport dynamics have been characterized for anion-selective nanofluidic membranes [72,73].

These examples span substantially different systems, from rigid inorganic nanogaps to biological pores and functionalized nanofluidic channels. Their common value for the present framework is therefore mechanistic rather than quantitative: they demonstrate that confinement can modify the influence of ion identity and surface chemistry, whereas the magnitude and even the form of this coupling remain system-dependent.

The confinement literature extends well beyond the systems discussed above. Carbon nanotubes provide a canonical one-dimensional platform in which water and ion transport are strongly affected by slip, dehydration, charge regulation, and entrance effects, while angstrom-scale slits and atomically thin membranes provide complementary two-dimensional and quasi-two-dimensional geometries. Electrified interfaces add an additional coupling between confinement, field, ion distribution, and water orientation. These classes are therefore important benchmarks for testing whether the proposed coupling relationships transfer across different confinement dimensionalities.

### 3.5. Ion-Series Reversal as an Indicator of Underlying Dependencies

The observations discussed in the two preceding subsections usually appear separately in the literature—either as isolated anomalies or as grounds for skepticism regarding the concept of a universal ion series. These observations are usually discussed in separate mechanistic contexts; here, we examine whether they can be usefully organized within a common conditional-coupling framework. Meanwhile, the accumulated data suggest that the reversal of the ion series should be viewed neither as an exception to the rule nor as a methodological artifact, but rather as an indication that ion action is conditioned by the status of another factor.

This conclusion is supported by a systematic analysis showing that reversal is triggered by a change in the sign of the surface charge, a transition of functional groups from hydrophobic to hydrophilic, an increase in salt concentration, or a change in pH [74]. These causes correspond to three of the five factors—surface chemistry, surface charge, and the thermodynamic or chemical environment—and do not include confinement. They therefore demonstrate that ion ranking can depend on the state of another control factor, but they do not by themselves establish coupling to all axes of the proposed map. Consequently, this phenomenon serves as an indicator rather than a definitive criterion and becomes significant only after these alternatives have been ruled out; assigning the effect to a specific edge remains a separate conclusion. Operationally, assignment of a reversal to a specific coupling edge requires a sequence of exclusion tests. First, the reversal should be reproducible for the same system and the same observable while a defined second factor is varied. Second, ion speciation and ion-pairing state should either remain unchanged across the reversal point or be independently quantified, because a change in chemical form can generate a new ordering without any change in factor coupling. Third, competitive adsorption and aggregation should be excluded by appropriate control measurements, since both can alter the apparent ion ranking through a change in the dominant population or interaction mechanism. Fourth, the reversal should not coincide with saturation of the measured response or with a transition to a different rate-limiting step. Finally, the factor proposed to constitute the second end of the coupling edge—for example, surface charge, confinement, or thermodynamic environment—should be independently characterized rather than inferred solely from the nominal experimental condition. Only when these conditions are satisfied should the reversal be assigned to a specific edge; otherwise, it should be reported more conservatively as evidence that the ion effect is conditional on a change in system state or mechanism. Early studies linked the reversal of the anion series to the combined influence of surface charge and polarity [75], while force microscopy demonstrated the decisive role of surface hydration [76].

When these alternative explanations are excluded or independently constrained, the interpretation allows diverse observations to be mapped onto specific couplings. For example, the edge connecting ion identity and the thermodynamic environment includes reversal upon pH change on silica [27], reversal of cation-specific effects on mica [77], reversal upon solvent exchange [30], and modulation of the series by osmolytes [29]. The alteration of ion effects upon the application of a field [31] relates to the interface between external influence and the nature of the ion, whereas the disappearance of selectivity in hydrophobic nanopores [78] pertains to the interface between the third and fourth categories. Cases involving intrinsic mechanisms also fall into this context: the reversed fractionation of carbonate and bicarbonate is explained by aggregation [79], while on copolymer brushes, the direction of the ion-specific effect depends on experimental conditions [80].

Hence the classification of systems based on ion-series behavior—subject to a caveat that fundamentally alters its application. Rank stability does not imply factor additivity. The ion order may remain constant even as effect magnitudes change drastically and differentially; this constitutes a strong interaction. Thus, rank stability merely indicates that the diagnostic procedure yielded no evidence of dependency; the question of additivity is resolved by comparing magnitudes rather than order, necessitating a factorial approach. Series-based diagnostics serve as a low-cost preliminary check with an asymmetric outcome: a change in order is informative and requires explanation, whereas stability is uninformative (Table 3). We emphasize this point because the alternative interpretation—mistaking order preservation for factor independence—appears in the literature and leads to erroneous conclusions. Determining the regime requires the independent variation of at least two factors, whereas many of the studies considered here vary only one. This limitation may contribute to the diversity of ion-ordering patterns reported in the literature (Figure 3).

A caveat is necessary to link the foregoing to the subsequent discussion of methodological contradictions. An inversion of the sequence established via one observable quantity need not be reproduced via another, as different methods are sensitive to different aspects of the water state. Therefore, it is correct to formulate the diagnostic criterion for a specific pair—comprising the system and the observable quantity—rather than for the system in isolation. A discrepancy between modes identified by different methods for the same system is informative in itself, indicating that the coupling affects structure and dynamics in distinct ways.

It is also useful to distinguish the proposed framework from explanatory schemes based on water-affinity matching. The water-affinity matching rule explains the preferential formation of ion pairs between ions of similar charge density [81] and remains a useful tool, yet it describes the properties of the ions themselves. In contrast, diagnostics based on sequence inversion characterize the system as a whole and are thus applicable to comparing dissimilar interfaces that share the same ion descriptors.

### 3.6. Limits of Controllability

A complementary question is where a factor ceases to exert a measurable influence. Here, it is important to distinguish between two phenomena often conflated in the literature (Table 4). Saturation indicates that the observable has reached a plateau as the factor is increased, whereas loss of response indicates that no measurable dependence on that factor is detected over the investigated range. Saturation has been observed for the second factor on model membrane surfaces, where charge-induced water alignment reaches a plateau above a certain threshold charge density, beyond which further increases in charge do not alter the orientation [82]. This result pertains to the orientational response and to lipid systems.

A more stringent constraint has been obtained for a different observable and a different class of materials. Quasielastic neutron scattering studies of hydrated films of carboxylated cellulose nanofibrils revealed that a nearly threefold change in carboxyl group density does not alter the mechanism of water dynamics; furthermore, the diffusion coefficient of the slow component remains close to the value observed for bulk water and for hydrated perfluorosulfonic acid membranes, regardless of the surface charge [6]. The issue here is not the attainment of a plateau at high charge levels, but rather the absence of any response—specifically a dynamic, rather than orientational, response—across the entire range investigated. The expectation that an increase in the density of charged groups should enhance water binding is not borne out at all in this system. We will revisit this case in Section 4, where it is placed within the broader context.

An “abolition” regime is identified regarding the nature of the ion, wherein ion selectivity completely vanishes within hydrophobic ion-exchange nanopores [78]. Here, strong confinement and pronounced wall chemistry coexist, and under these conditions, the system ceases to distinguish between ion types.

The implication for future design principles is as follows. Control axes exhibit saturation and abolition regions; beyond these limits, further intensifying the influence does not alter the water state, with the location of these regions depending on the specific observable quantity under consideration. Consequently, optimal material performance does not necessarily correspond to the maximum value of any given factor, and the notion that more strongly bound water always equates to superior performance lacks a general basis.

## 4. Structure and Dynamics

### 4.1. Why the Distinction Is Essential

As defined in Section 2.3, orientational order, hydrogen-bond strength, and water dynamics are distinct observables. The question addressed here is whether structural and dynamic responses covary when the same control parameter is changed.

### 4.2. Established Facts Regarding Structure

Charge-dependent orientational ordering at aqueous interfaces is well-established and has been reviewed extensively [5,83]. Nonlinear optical measurements directly demonstrate charge-dependent orientation, including reversal with charge sign [83]. These observations concern orientational order and do not by themselves establish corresponding changes in water dynamics.

For the subsequent discussion, the only important point is that all the cited evidence relates to orientational characteristics and was obtained primarily through symmetry-based methods; no conclusions regarding dynamics can be drawn from it.

### 4.3. Dynamics and the Scope of the Knowledge Gap

Within the literature surveyed here, the principal gap is not the absence of dynamic measurements but the limited number of matched studies that probe structural and dynamic observables while varying the same control parameter.

It is known that the vibrational relaxation of interfacial water differs from that of bulk water and depends on the sign of the surface charge. For positively charged monolayers, the dynamics resemble those of bulk water and the air–water interface, whereas for negatively charged monolayers, the dynamics differ—a phenomenon attributed to strong hydrogen bonding with the negatively charged headgroup moiety [84,85]. A general overview of ultrafast dynamics at aqueous interfaces is available in the literature [86], as is a summary of water dynamics in the hydration shells of biomolecules [87].

Recent studies have extended measurements of water dynamics from neutral interfaces to charged and electrified systems, including graphene, micelles, electrodes, and metal surfaces [88,89,90,91,92,93,94]. However, these studies involve different observables, surface chemistries, geometries, and time windows, and rarely combine structural and dynamic measurements under controlled variation of the same parameter. The gap therefore lies not in a lack of measurements but in their fragmented nature. Accordingly, differences between these studies cannot be interpreted solely as differences in water dynamics: they also reflect distinct surface chemistries, geometries, ionic environments, and instrumental time windows. Their comparison is therefore qualitative unless measurements are performed under matched conditions. It is precisely this latter element—rather than the dynamic data themselves—that is missing for classifying systems according to the categories introduced below.

### 4.4. Structure Does Not Necessarily Predict Dynamics

Representative studies demonstrate that structural and dynamic responses can become decoupled under specific experimental conditions.

The case of zwitterionic lipid monolayers is particularly illustrative. Reversing the order of the phosphate and choline groups in the headgroup alters the average orientation of interfacial water by approximately 180 degrees—an extreme structural change. Yet, the vibrational dynamics of the lipid-associated interfacial water remain independent of this orientation [95]. The structure undergoes a complete reversal, while the dynamics show no response whatsoever. The second case concerns polymer films and has already been discussed in Section 3.6. A nearly threefold change in the density of carboxyl groups in hydrated cellulose nanofibril films does not alter the mechanism of water dynamics [6]. Although the surface chemistry changes substantially, the measured dynamic characteristics remain unchanged.

At the silica–water interface, increasing surface charge shortens the population lifetime of the hydrogen-bonded OH stretch [96]. This quantity reflects vibrational energy relaxation rather than translational or rotational molecular mobility and should therefore not be used as evidence of counter-directional structure–mobility behavior. We consequently do not classify this example as a structure–dynamics inversion.

The inverse approach—using dynamics to infer structural heterogeneity [97]—presupposes the existence of a correspondence; however, the cited examples demonstrate that this correspondence must be verified for each specific system. Several studies have measured structural and dynamic observables within the same system [98,99,100,101,102,103,104,105], but few vary a single factor under matched conditions. Therefore, simultaneous measurement alone is insufficient to establish structure–dynamics coupling.

### 4.5. Degree of Correspondence as a System Characteristic

The cases discussed allow for an ordering in which the correspondence between structure and dynamics is treated as an intrinsic system characteristic. Two limiting behaviors observed in the literature are described below. These do not represent discrete natural classes; rather, continuous transitions between them should be expected. They serve as useful reference points for positioning specific systems, rather than as an exhaustive typology (Table 5, Figure 4).

Available studies demonstrate that coupled and decoupled relationships between structural and dynamic observables can occur under different conditions. However, the current literature does not permit a reliable comparison of how frequently these behaviors occur, because paired measurements of structure and dynamics under controlled variation of a single factor remain limited. Moreover, the literature selection used in this review was not designed to quantify their relative prevalence. We therefore treat the two cases as limiting conceptual behaviors rather than as evidence-ranked categories; the examples in Table 5 illustrate their mechanistic possibility, not their frequency across the field.

This distinction entails a requirement regarding the formulation of results. A conclusion concerning the state of interfacial water derived from a structural method cannot be extrapolated to dynamic characteristics without specifying the case to which the system belongs, and vice versa. This requirement is essentially the same as the requirement to specify the observable—as formulated in Section 2.3—but it adds the proviso that extrapolation between structure and dynamics constitutes a distinct assumption rather than a self-evident step.

There are three implications here. First, the caveat in Section 3.5—stating that a “regime” is defined for a specific pair consisting of a system and an observable—is substantiated: “decoupling” implies that varying a single factor may alter the ordering of ions according to a structural observable while leaving the order unchanged according to a dynamic one; thus, a divergence in regimes between methods serves as an indicator of decoupling.

## 5. What Measurements Can—And Cannot—Inform Materials Design

### 5.1. Basis of Classification

For materials design, the central methodological question is not which technique is most sophisticated, but which physical property it actually constrains. Different methods probe different observables, spatial regions, and timescales; therefore, they cannot be used interchangeably as descriptors of a single “water state”. The classification below is retained only to identify which design-relevant quantities each method can and cannot support.

This classification clarifies why methods yield different observables and which combinations can resolve specific discrepancies.

### 5.2. Families of Methods

Symmetry-based methods exploit the fact that a second-order nonlinear response arises only when centrosymmetry is broken—a condition that occurs at the interface between two media. This category encompasses sum-frequency generation, second-harmonic generation, and second-harmonic scattering, with the latter extending the approach to particles and droplets in the bulk [106]. This family of techniques provides information on orientational order and surface potential, while signal decomposition allows the response of the interfacial layer to be distinguished from that of the more distant region [107]. Symmetry-based methods report orientational order and interfacial electric response but do not directly provide water mobility or a complete structural description [108,109]. Mechanical methods provide force or density information near the interface but do not directly resolve dipole orientation [110]. NMR and neutron-scattering methods provide relaxation, exchange, and mobility information, but usually with limited spatial localization relative to the interface.

Electronic methods utilize photoabsorption and scattering cross-sections. Photoelectron spectroscopy provides core-level energies sensitive to the local environment and potential, while X-ray reflectometry yields the electron density profile along the surface normal—a technique used to establish the existence of water density oscillations near mica surfaces [9]. A limitation is the signal acquisition time, which renders dynamic characteristics inaccessible.

Collective polarization methods measure the aggregate polarization response of the medium. A notable example is the direct determination of the dielectric permittivity of water in nanometer-scale gaps, which yielded anomalously low values [2]. This family provides a quantity that appears in expressions describing ion–surface interactions but does not distinguish between individual molecular positions; the bulk of its applications concerns the hydration water of biomolecules—such as lysozyme, lipid bilayers, protein dynamics, and DNA duplexes [111,112,113,114]—as well as complex dispersed systems [115]. To date, however, the family has not been systematically applied to water at solid surfaces.

Linear vibrational spectroscopy reports hydrogen-bond-sensitive spectral changes but may contain substantial bulk contributions [116]. Probe-based methods offer spatial localization but may perturb the local environment being measured [117,118].

Computational approaches occupy a unique position because they provide access to any quantity, albeit within the limits of the chosen model’s accuracy. Classical molecular dynamics is constrained by the quality of the force field; most fields are designed to accurately describe water–ion interactions but perform significantly worse for ion–ion interactions—a factor that becomes critical at high concentrations and necessitates the development of force fields incorporating many-body contributions [119]. First-principles and density-functional-theory-based molecular dynamics avoid empirical force-field parametrization but introduce their own approximations, including the choice of exchange-correlation functional, finite system size, limited trajectory length, and incomplete treatment of nuclear quantum effects. They should therefore be regarded as complementary to, rather than as simply overcoming, classical force-field limitations, while also being substantially more computationally demanding.

### 5.3. Inherent Limitations of the Families

A summary of inaccessible characteristics is provided below. The third column should be interpreted not as a list of flaws, but as a consequence of the type of coupling involved (Table 6). Only the limitation listed in the first row is absolute—stemming from a selection rule—whereas the others reflect a weak coupling between the measured quantity and the target characteristic and are, in this sense, fundamentally surmountable.

### 5.4. Chain of Assumptions

For many methods, the quantity of interest is not measured directly but is inferred from the primary experimental signal through a model-dependent transformation. A chain of transformations—each relying on specific assumptions—intervenes between the raw signal and the final figure (Table 7). It is precisely this chain that gives rise to the model-extraction discrepancies discussed in Section 6.5.

Model dependence is particularly important when raw signals are converted into quantities such as surface potential or fractions of distinct water states. For design purposes, such derived quantities should therefore be treated as model-dependent descriptors rather than direct observables, and conclusions should be cross-checked where possible using an independent measurement [120,121,122,123,124,125,126,127].

### 5.5. Combinations That Resolve Discrepancies

Distinguishing between these families makes it possible to identify which combination of methods can resolve a specific type of discrepancy (Table 8).

Several ambiguity classes can be resolved without introducing new water-sensitive techniques. Independent measurements of surface state, solution composition, and sample history are often sufficient to determine whether an apparent difference reflects the material itself or the measurement protocol [128]. The corresponding experimental checks are discussed further in Section 5.5 and Section 6.4, where independent characterization of surface state and solution composition is identified as the key safeguard against hidden variation of coupled factors.

## 6. Reconciling Measurements for Materials Design

### 6.1. Discrepancies as a Subject of Analysis

For materials design, an apparent disagreement between methods becomes important only if it changes the inferred design variable or the expected material response. We therefore classify recurrent discrepancies according to whether they arise from the observable, timescale, probed region, sample state, preparation history, or model used to extract the reported quantity.

### 6.2. What the Methods Actually Measure

None of the methods currently in use measures the state of interfacial water per se. Each records a specific projection of that state, determined by the physical nature of the response, the temporal sensitivity window, and the spatial region from which the signal is collected (Table 9).

Measurements can be compared directly only when their observables and sensitivity windows overlap sufficiently for the design question being addressed.

### 6.3. Discrepancies That Are Not Discrepancies

Two methods may report different trends simply because they probe different properties [107]. A structural observable should therefore not be used as a proxy for mobility, and vice versa, unless the correspondence has been established for that system.

The second type arises from differences in time windows. A water molecule that appears stationary within a picosecond window may prove mobile within a nanosecond window; thus, the experimentally assigned fraction of bound water is not an intrinsic, method-independent quantity but depends on the observation timescale. Discrepancies regarding the magnitude of this fraction between spectroscopic and relaxation methods are to be expected, and their absence would actually require an explanation. Only values obtained within overlapping windows are comparable; comparing vibrational spectroscopy results with those from nuclear magnetic resonance requires an explicit model of the distribution of timescales.

The probed volume may itself change with ionic strength or measurement geometry. Consequently, an apparent concentration dependence can partly reflect a change in sampling depth rather than a change in the material state. This must be excluded before using the measured trend as a design rule.

### 6.4. Discrepancies Regarding the System, Not the Measurement

A fourth type of discrepancy can arise when the surface state is not measured independently but is instead inferred from the bulk composition of the solution. Surface chemistry and surface charge are coupled, and the surface pKa value itself depends on the structure of the interface [15]; consequently, two samples at the same bulk pH may nevertheless exhibit different surface charge states. The resulting discrepancy reflects a genuine difference between the systems rather than a difference in measurement methods; resolving it requires an independent determination of the degree of protonation rather than increased spectroscopic precision.

A fifth type arises from unintended variation of a coupled factor. In a real experiment, attempting to alter one factor while keeping others constant typically leads to a change in an unaccounted-for factor, because the accessible parameter space is not a simple Cartesian product of the individual factors. Replacing a salt to alter the nature of an ion simultaneously changes the ionic strength; adjusting pH to control charge simultaneously alters the donor–acceptor balance of surface groups; and distinguishing the behavior of water in the Stern layer from that in the diffuse layer requires a specially designed series of measurements with varying salt concentrations [129]. Comparisons ostensibly made under identical conditions are often, in fact, made under differing ones.

A sixth type arises from differences in sample history. Fluid flow along a solid surface reversibly alters its interfacial chemistry [32], while hydration cycles induce hysteresis in the wetting of nanochannels [61]. Samples subjected to different preparation procedures constitute distinct systems, and specifying the solution composition does not fully define the state of the system. This is particularly evident on hydrophobic surfaces, where trace impurities can determine the observed charge and thereby give rise to discrepancies attributed to the methodology [130]. Moreover, flow alters the forces between charged solid surfaces and displaces the double layer from equilibrium [131].

### 6.5. Discrepancies Regarding the Extracted Quantity

The seventh type occupies a unique position, as it relates neither to the measurement itself nor to the system, but rather to the conversion of the signal into the quantity being reported. The intensity of the nonlinear optical response is converted into a surface potential using double-layer models—the very models whose applicability is the subject of debate. The reported quantity therefore depends on the chosen model, and a discrepancy between studies may actually reflect a difference in the extraction models used, even when the measurements themselves are identical.

Separating contributions of different orders presents an additional challenge. Assessing the effect of salt on water structure near a charged solid surface requires distinguishing between second- and third-order contributions [120], while separating the contribution of surface structure from that of surface charge requires comparing two scattering methods [121]. A comparison of non-resonant second-harmonic generation and resonant sum-frequency generation on the same system revealed that the extracted quantities agree only under specific assumptions [125], and a quantitative analysis of the surface potential at the electrolyte–air interface required a separate modeling approach [124]. The broader question of whether the interfacial potential is measurable in principle remains a subject of discussion [127].

Also falling into this category are discrepancies between calculations and experiments arising from force-field effects. Most force fields are designed to accurately describe water–ion interactions but are significantly less accurate in describing ion–ion interactions—a factor that becomes critical as concentration increases—leading to the separate development of force fields that incorporate many-body contributions [119]. A discrepancy between calculation and experiment at high ionic strength does not necessarily indicate an incorrect physical model. Moreover, the result depends on the assumed thickness of the Stern layer—a parameter factored into the calculation of the diffuse layer capacitance that is typically chosen rather than measured [132]—whereas theories that explicitly account for water yield different predictions regarding ion specificity compared to continuum models [133].

### 6.6. An Illustrative Case of Method-Dependent Interpretation

The discussion of ion accumulation at water–air and water–hydrophobic interfaces illustrates how apparently conflicting conclusions may arise from differences in both the observable and the interface being examined. Molecular-level studies indicate that excess hydrated protons can be significant at the air–water interface [134], whereas hydroxide stabilization has been demonstrated at hydrophobic monolayers [135]; moreover, a spectroscopic observable may remain insensitive to part of the underlying interfacial response [107]. These results should therefore not be treated as directly contradictory without accounting for the probed spatial region, measured quantity, and surface state. This example illustrates why mechanistic interpretations should not be transferred between interfaces solely on the basis of an apparently similar macroscopic response.

### 6.7. Minimum Set of Data for Comparability

The practical outcome of this section is a list of data points that, when reported, render results from different studies comparable (Table 10). This does not entail additional measurements beyond those already performed but merely requires their documentation.

A structured reassessment of the core studies used in the coupling map and pathway analysis showed that the seven reporting elements listed in Table 10 are not documented with equal consistency. The measured observable and principal experimental variables are generally identifiable, whereas independent characterization of surface state, explicit definition of the probed spatial or temporal window, extraction-model assumptions, and sample history are less consistently reported. Because the studies included in this review constitute a purposefully selected mechanistic literature corpus, these observations are not converted into prevalence estimates. They instead identify the reporting dimensions that most directly limit cross-study comparison within the evidence considered here.

## 7. From the State of Water to Material Function

### 7.1. Three Interpretive Pathways Linking Water State to Material Function

To distinguish levels of evidence, we use three categories throughout this section. “Established observations” refer to relationships directly demonstrated in the cited experimental or computational studies. “Interpretations” refer to our synthesis of multiple observations into a common mechanistic pathway. “Design principles” are forward-looking implications derived from that synthesis and should be regarded as testable propositions rather than universally established rules. The three pathways introduced below therefore constitute an interpretive framework for organizing existing evidence; they are neither mutually exclusive nor assumed to be universally dominant across entire material classes. We use the term “energetic pathway” to group studies in which changes in interfacial-water state are associated with changes in the free-energy landscape of an elementary step, such as solute adsorption, ion entry into a pore, binding to a site, or transition-state formation.

The “transport pathway” groups observations in which water dynamics are linked to mobility-related material properties, including diffusion, conductivity, flux, and relaxation behavior. Dynamic quantities are the determining factors, and, as shown in Section 4, these do not stand in a one-to-one correspondence with structural quantities.

The “occupancy and screening pathway” groups observations in which hydration-shell volume, dielectric response, or screening modifies the effective geometry or interaction range within the material. The extent of the ordered region and its dielectric properties—rather than energy or mobility—serve as the determining factors.

The examples discussed below differ substantially in geometry, surface chemistry, electrolyte composition, and operating regime. They are therefore used to identify recurring mechanistic patterns rather than to imply quantitative equivalence between material classes.

### 7.2. The Energetic Pathway

The essence of the energetic pathway is encapsulated in the premise that water is a third participant in the process, rather than merely the medium in which the process takes place. Sorption, ion entry into the pore, and binding to a site are described not by a surface–solute pair but by a triplet in which water contributions enter with opposite signs. Within this interpretation, the functional outcome depends on the balance between these energetic contributions rather than on water-binding strength alone. These observations indicate that stronger surface binding does not, by itself, guarantee improved material performance.

Quantitative evidence is most clearly manifested in ion transport across an interface, where the hydration number—specifically, the number of water molecules in the first shell—determines ion mobility along the surface [4]. Here, the state of water enters the function not indirectly, but as a direct parameter of the barrier. A related result has been obtained regarding binding selectivity at aqueous interfaces, where the determining factor is the combination of molecular recognition and a mismatch in the hydration energies of the partners [136]. Both studies are theoretically grounded in the concept of the preferential formation of ion pairs between partners with similar charge densities [81]; however, in interfacial systems, the role of the partner is played by a surface functional group whose effective charge density itself depends on the state of the interface.

For reversible sorption, stronger binding may improve uptake while hindering regeneration, implying that optimal binding strength may be intermediate rather than maximal [137].

A practically significant consequence arises in two-stage processes with conflicting requirements. In sorption materials, the same energetic contributions that favor uptake may hinder subsequent release during regeneration. For systems in which uptake and regeneration impose opposing requirements, an intermediate binding strength may therefore be preferable to maximizing binding strength alone. This factor is crucial for evaluating separation systems, where the choice between extraction, sorption, and membrane separation is dictated by a combination of requirements rather than a single parameter [138].

### 7.3. Transport Pathway

The transport pathway appears to be the most direct, as it links water and ion mobility to conductivity and flux through the material. It is also the pathway most commonly invoked in membrane design. However, it is precisely here that the degenerate link discussed in Section 4 is located.

As discussed in Section 4.4, cellulose nanofibril films provide an example in which charged-group density is weakly coupled to water dynamics [6].

This degeneracy, however, is not universal; its boundaries are defined by geometry. In confined geometries, the transport pathway restores density dependence, with both the type of functional group and the surface coverage acting as control factors: in transition-metal carbide channels, increasing fluorine group coverage suppresses the transport of both cations and anions [58]; graphene functionalization governs the structure and dynamics of confined electrolytes [59]; and in multilayer membranes, functional groups simultaneously determine water flux and ion rejection [60]. Taken together, these observations suggest that the strength of the relationship between surface functionality and transport is itself dependent on confinement conditions. In the polymer-membrane examples considered here, this relationship appears comparatively strong [139,140], and the discrepancy with cellulose films requires an explanation that is currently lacking.

### 7.4. Confinement and Screening Pathway

The third pathway operates not through energy or mobility, but through volume and the range of interactions. The hydration shell occupies part of the channel lumen, causing the effective pore aperture to differ from the nominal one, while the ordered water region near the surface alters the medium’s dielectric response.

Direct measurements in nanometer-scale gaps have reported a strongly reduced out-of-plane dielectric component [2], while confined water can exhibit anisotropic dielectric response [63]. These observations imply geometry-dependent modification of electrostatic interactions rather than a uniform scalar renormalization of all interactions. Surface-force measurements have reported anomalously long decay lengths in concentrated electrolytes [53]. However, interpretation of these measurements as a universal bulk screening length remains debated: theory and simulations do not consistently reproduce anomalous underscreening under comparable conditions, and its microscopic origin remains unresolved. Nuclear magnetic resonance studies of nanoconfined electrolytes have revealed a breakdown of local electroneutrality [52], while the selectivity of a charged nanopore is determined by the combined action of several physical fields [57].

The functional application of this pathway is most clearly demonstrated in electrokinetic converters, where the output characteristic is determined by the ratio of the charge-separation zone’s extent to the channel size, and where overcoming the limitation imposed by the Debye length enables the extraction of electrical energy from natural water flow [141]. Here, the state of the water neither facilitates nor hinders a specific process but constitutes the device’s working medium itself.

### 7.5. Provisional Mapping of Pathways Across Representative Systems

The assignment of a predominant pathway in Figure 5 represents our interpretation of the balance of evidence within the systems considered, rather than an experimentally established intrinsic property of a material class. Where the available evidence does not discriminate among pathways, no predominant assignment is made. Representative evidence relevant to this mapping includes studies of electrified interfaces, in which interfacial-water properties are linked to electrochemical response [27,142,143,144], as well as studies of bipolar nanopores, lipid monolayers, hydrogels, and functionalized membranes that illustrate pathway-relevant hydration, transport, and ion-specific effects [69,145,146,147]. These examples support the mechanistic interpretation shown in Figure 5 but do not establish a universally predominant pathway for the corresponding material classes.

### 7.6. Antifouling Coatings as an Unresolved Case

Antifouling coatings provide a useful counterexample to pathway assignment because the currently available evidence supports several competing mechanisms without establishing their relative contributions.

It is generally accepted that the resistance of hydrophilic and zwitterionic coatings to protein adsorption is ensured by a barrier of tightly bound water. However, this explanation masks three distinct mechanisms corresponding to the three pathways. Studies on the interfacial hydration of antifouling polymers describe the effect in terms of water binding strength—that is, energetically [148,149]. Research into the three-dimensional structure of the hydration layer on fouling-resistant nanoparticles describes it as an extended ordered region, invoking the occupancy pathway [110]; selective protein adsorption on saccharide monolayers is explained in similar terms, via hydration repulsion [150]. Finally, it has been shown that antifouling performance also depends on ligand dynamics [151], a finding that points to the transport pathway. It should be added that ions and surface potential act jointly on such a coating [152], that the nature of the anion significantly alters the hydration and layering of polyzwitterionic brushes [153], and that hydration can be detected directly on the working surface [154]. These explanations are not mutually exclusive. The available evidence is compatible with contributions from all three pathways, although their relative importance has not been established. However, a discriminating experiment capable of establishing their relative weight has not yet been conducted, and the predictions of the three pathways diverge. The energetic pathway predicts a correlation between stability and water-binding strength; the occupancy pathway predicts a correlation with the extent of the ordered region; and the transport pathway predicts a correlation with relaxation times. Designing an experiment in which these three quantities are varied independently constitutes a clearly defined challenge, and its resolution would have significance far beyond the field of protective coatings.

### 7.7. Feedback of Function on the State of Water

The foregoing describes a transition in one direction. However, the material in operation alters the state of interfacial water established during its characterization, which limits the applicability of the entire framework.

This is most directly evidenced by the fact that fluid flow along a solid surface reversibly alters its interfacial chemistry [32]. The surface in an operating device is not in the same state as when it was measured at rest. We must also consider the dependence on history, as hydration cycles and wall polarity determine the impregnation hysteresis in silica nanochannels [61]—meaning that the current state is determined by more than just current conditions.

This gives rise to two limitations. Characteristics measured at equilibrium should not be assumed to remain valid under operating flow conditions without independent verification, because flow itself can alter interfacial chemistry. The range of flow conditions over which equilibrium-derived descriptors remain applicable therefore requires system-specific assessment. Materials operating cyclically—including regenerable sorbents and membranes subject to periodic washing—require the state to be treated as a function of history; this falls outside the scope of the present discussion but is identified in Section 10 as an area for future work.

### 7.8. Why Comparisons Across Domains Have Not Previously Aligned

The distinction between pathways provides one possible explanation for the difficulty of reconciling results across different application domains. The same nominal intervention can produce different functional consequences because it may affect different water-related observables. For example, a surface modification that strengthens interfacial hydration may reduce water or ion mobility along a transport pathway while simultaneously increasing hydration-mediated exclusion along an occupancy/screening pathway. These effects should be verified independently because the relevant observables need not covary.

## 8. A Unified Picture: Factors, Their Interconnections, and the Causal Chain

### 8.1. The Causal Chain and Its Non-Uniformity

The material presented in the preceding sections can be synthesized into a coherent picture centered on a causal chain leading from the interface—via the state and dynamics of the water—to transport processes, and ultimately to material function. A link may be described as “dense” when distinct input states correspond to distinguishable output states, and “sparse” when noticeably different input states yield indistinguishable outputs. A sparse link does not break the chain but renders it impassable in the reverse direction; reconstructing the surface chemistry from the observed function proves impossible, even with full knowledge of the intermediate states.

This remains a qualitative heuristic; quantitative metrics of link strength require controlled datasets that are currently scarce.

### 8.2. The Factor Space Is Constrained and Non-Orthogonal

The second element of the picture concerns the input component. In practical terms, the five control factors cannot generally be optimized one at a time. Changing pH may alter both surface charge and surface chemistry; changing the electrolyte changes ion identity together with ionic strength; and introducing confinement can modify dielectric response and ion distributions. Materials optimization should therefore either vary coupled factors factorially or independently measure the accompanying change in the second factor.

The third element is the method of identifying underlying dependencies based on observed behavior. Ion-series reversal provides a conditional diagnostic of factor coupling only when the exclusion criteria defined in Section 3.5 are satisfied.

The conclusion drawn from this interpretation is asymmetric, and it is crucial to emphasize this asymmetry. A reversal in ranking indicates that sequential optimization may miss the optimum because the preferred condition depends on the level of the second factor. The control-parameter value at which reversal occurs identifies a condition-specific transition point, but it should not be interpreted as a quantitative measure of coupling strength without an explicit model. While rank stability does not *forbid* sequential optimization, it does not *guarantee* its validity either; such validation requires demonstrating that the interaction is negligible in terms of effect magnitude—something that rank stability alone cannot establish. The suppression of selectivity implies that further optimization of electrolyte composition based on the observed quantity would be futile; however, since there are multiple potential causes for this suppression, one must rule out the mutual compensation of mechanisms and insufficient method sensitivity before concluding that saturation has occurred.

The framework should therefore be regarded as qualitative and hypothesis-generating rather than as a quantitatively validated predictive model. The coupling map summarizes relationships supported by the literature surveyed here, but it does not assign numerical coupling strengths or predict effect sizes across material classes. Its testability lies instead in the experimental predictions it generates: a proposed coupling should persist when two factors are varied independently under matched conditions, whereas an apparent relationship should disappear when the alternative mechanism responsible for it is controlled. Quantitative parameterization of these relationships requires factorial datasets that are not yet available for most of the systems considered here.

## 9. Proposed Design Principles and Their Limits

### 9.1. Why Characterization Does Not Automatically Translate into Control

The methodological discussion in Section 5 and Section 6 has a direct design consequence: a material should be optimized against the observable that is mechanistically linked to the target function, rather than against whichever descriptor is most convenient to measure. A structural, dynamic, dielectric, or surface-potential descriptor is useful only when the corresponding link to function has been established for the system under study. This leads to four practical rules. For surface-functionalized materials, do not prescribe pH, surface chemistry, and charge as independent design variables without measuring the actual surface state. For reversible sorbents, do not maximize binding strength without considering regeneration. For membranes and nanopores, do not transfer trends measured at extended interfaces directly to confined geometries without revalidation. For antifouling coatings, the optimization descriptor should be selected according to the mechanism being tested: water-binding strength for an energetic mechanism, hydration-layer extent for an occupancy-based mechanism, and relaxation or ligand-dynamic times for a transport-related mechanism. A generic increase in “hydration” is not, by itself, a sufficient design criterion.

The literature on interfacial water has amassed a wealth of characterization data but often provides limited direct guidance for materials design. The principles proposed below are not presented as universal design rules. They are hypotheses and decision criteria derived from the evidence synthesized in Section 3, Section 4, Section 5, Section 6, Section 7 and Section 8 and should be validated for each material class and operating regime. This disconnect stems not from a lack of data, but from the fact that characterization and design address different questions. Characterization asks about the state of water in a given system, whereas design asks what must be altered to achieve a desired function. The latter question requires knowledge of available interventions, their independence, and the expected response—knowledge that cannot be directly derived from characterization data alone.

Because surface chemistry, charge, and pH are coupled, design specifications should not treat them as independently selectable variables [14,15]. For example, if membrane functionalization is intended to increase surface charge, the resulting charge should be measured under the intended operating pH rather than inferred from functional-group density alone. Otherwise, an apparent improvement attributed to functionalization may instead reflect a change in protonation state.

Designing a function via an intermediate water-state characteristic is feasible only when all links in the chain connecting the control variable to the function are sufficiently robust. Upon encountering a degenerate link, the designer faces a situation where the chosen control route may be ineffective even if the intermediate quantity is measured with greater precision. Optimization through an intermediate water-state descriptor is meaningful only when its links to both the control variable and target function are established. If a weak or degenerate link is identified, changing the control variable or the intermediate characteristic may be more effective than simply increasing measurement precision.

The optimization strategy depends on whether the effect of one factor is conditioned by the state of another, and the conclusion in this regard is asymmetric. A reversal of the ion series implies that the effect of one factor depends on the level of another; consequently, a sequential variation approach is likely to miss the optimum. A suppression of selectivity indicates that the factor in question elicits no response regarding the observed parameter, meaning that optimization efforts are justified only after the method’s sensitivity has been verified. Conversely, rank stability offers no definitive insight; it merely signifies an absence of evidence, and concluding that additivity exists requires a separate factorial experiment comparing the magnitudes of effects rather than their order. The widespread use of single-parameter optimization may contribute to the limited transferability and reproducibility of reported optima when processes are scaled up or transferred between systems. Accumulated data contradict the common assumption that intensifying the influence of any given factor invariably improves performance. These observations suggest that identifying a saturation region before further increasing a control parameter may prevent ineffective optimization.

### 9.2. Specification and Transfer

Selectivity should be reported together with the environmental range over which it was established, because ion rankings can change with pH, ionic strength, and confinement [27,56,74].

Transfer of design rules from extended to confined interfaces requires revalidation because confinement changes dielectric response, screening, and ion distributions [2,53,57]. Direct testing of transferability between chemically distinct systems would require paired interfaces that differ in ionizable-group chemistry and matrix rigidity while retaining comparable charge characteristics and being examined under matched conditions [155]. Such a comparison is experimentally feasible but has not yet been systematically implemented (Figure 6).

## 10. Open Questions

Established areas of research—such as measurements under operating conditions, the non-equilibrium double layer, force fields for concentrated solutions, and density functional theory-based dynamics—are covered in the literature [5] and are not repeated here.

A first, relatively low-cost priority is to improve reporting according to the checklist provided in Section 6.7. This checklist does not call for additional measurements but merely requires a description of those already performed; nevertheless, adhering to it would improve comparability for discrepancies classified as types one, two, and three. The second approach involves independently determining the surface protonation state while simultaneously measuring the state of the water; since surface chemistry and charge are linked, the absence of such a determination makes it impossible to distinguish between discrepancies caused by methodological differences and those arising from actual differences in sample states. The third approach entails sequentially measuring structural and dynamic characteristics on a single sample—without remounting it—while varying a single parameter under controlled conditions. Paired structural and dynamic measurements of this kind remain limited; such measurements are needed to determine how frequently for the two limiting behaviors identified in Section 4.

A future quantitative extension of this analysis should assess reporting completeness across a prospectively defined and statistically representative literature sample. Such an audit would determine whether the qualitative reporting gaps identified in Section 6.7 are characteristic of the wider field and would provide a quantitative baseline for future comparisons.

Within the literature surveyed here, links involving surface chemistry, charge, and ion identity are represented by more examples than confinement-related links. A priority task is to reproduce the effects established for confined geometries across several systems belonging to different classes. The task of disentangling interfacial contributions from those arising from nanoconfinement is already being addressed for specific systems [62], and a systematic approach to this problem is the logical next step.

The question of whether the list of governing factors is exhaustive also remains open. Establishing causality requires the independent variation of at least two factors, whereas controlled two-factor studies appear uncommon within the literature surveyed here. Consequently, the absence of a correlation in currently available data does not necessarily imply the absence of a physical link.

Decomposing the transition to a specific functional state into three dominant pathways gives rise to a class of problems concerning the relative weight of these pathways. The case of antifouling coatings—discussed in Section 7.6—is particularly suitable for such analysis; different studies support distinct mechanistic interpretations, but we did not identify a discriminating experiment in the literature surveyed here that establishes their relative contributions. What is needed is a set of surfaces in which water-binding strength, the extent of the ordered region, and relaxation times vary independently of one another.

While the need to describe non-equilibrium states is acknowledged, the material reviewed here helps refine the problem formulation. One key question is whether the characteristic behavior of the ion series persists during flow, particularly if the flow along the surface reversibly alters its interfacial chemistry [32]. Another is whether the hysteresis observed during hydration cycles [61] reflects a dependence of the state on its history—a scenario that would necessitate introducing a state variable alongside the governing factors. Both questions can be investigated using existing experimental techniques.

## 11. Conclusions

Interfacial water is best described through specific observables rather than a single notion of “structure”, and its state emerges from coupled rather than independent surface, ionic, confinement, and environmental factors. Ion-series reversal can provide a useful screening indicator of conditional factor coupling, but only after alternative chemical, aggregation, kinetic, and measurement-related causes have been excluded.

We interpret the available evidence through recurrent energetic, transport, and occupancy/screening pathways linking interfacial water to material function. These pathways and the resulting design implications should be regarded as an organizing framework and a set of testable propositions rather than universal rules. Future progress will depend particularly on controlled multi-factor experiments, matched structural–dynamic measurements, complete reporting of experimental conditions, and validation under operating conditions.

## Figures and Tables

**Figure 1:**
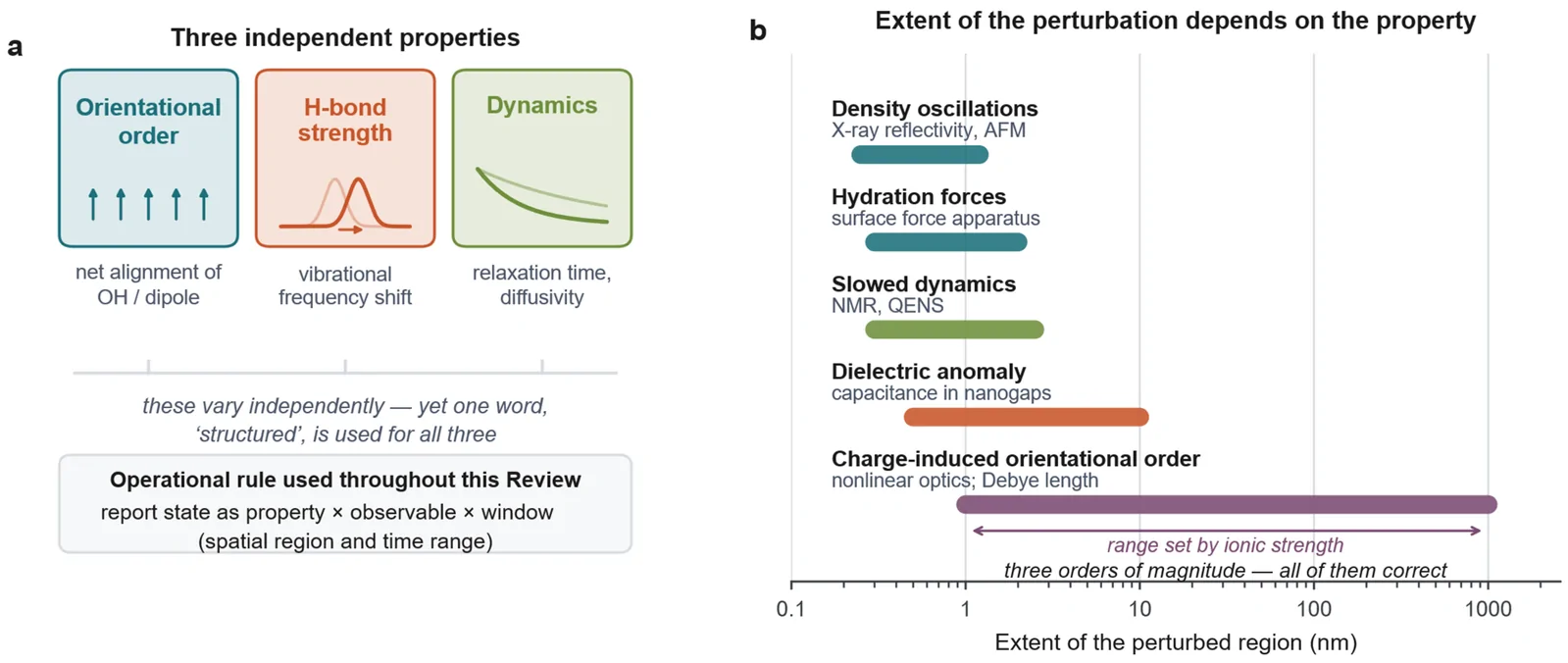
Characterization of interfacial water through distinct structural and dynamic observables. The bands indicate the approximate spatial range over which each property differs from bulk behavior: (**a**) three independent properties; (**b**) extent of the perturbation depends on the property.

**Figure 2:**
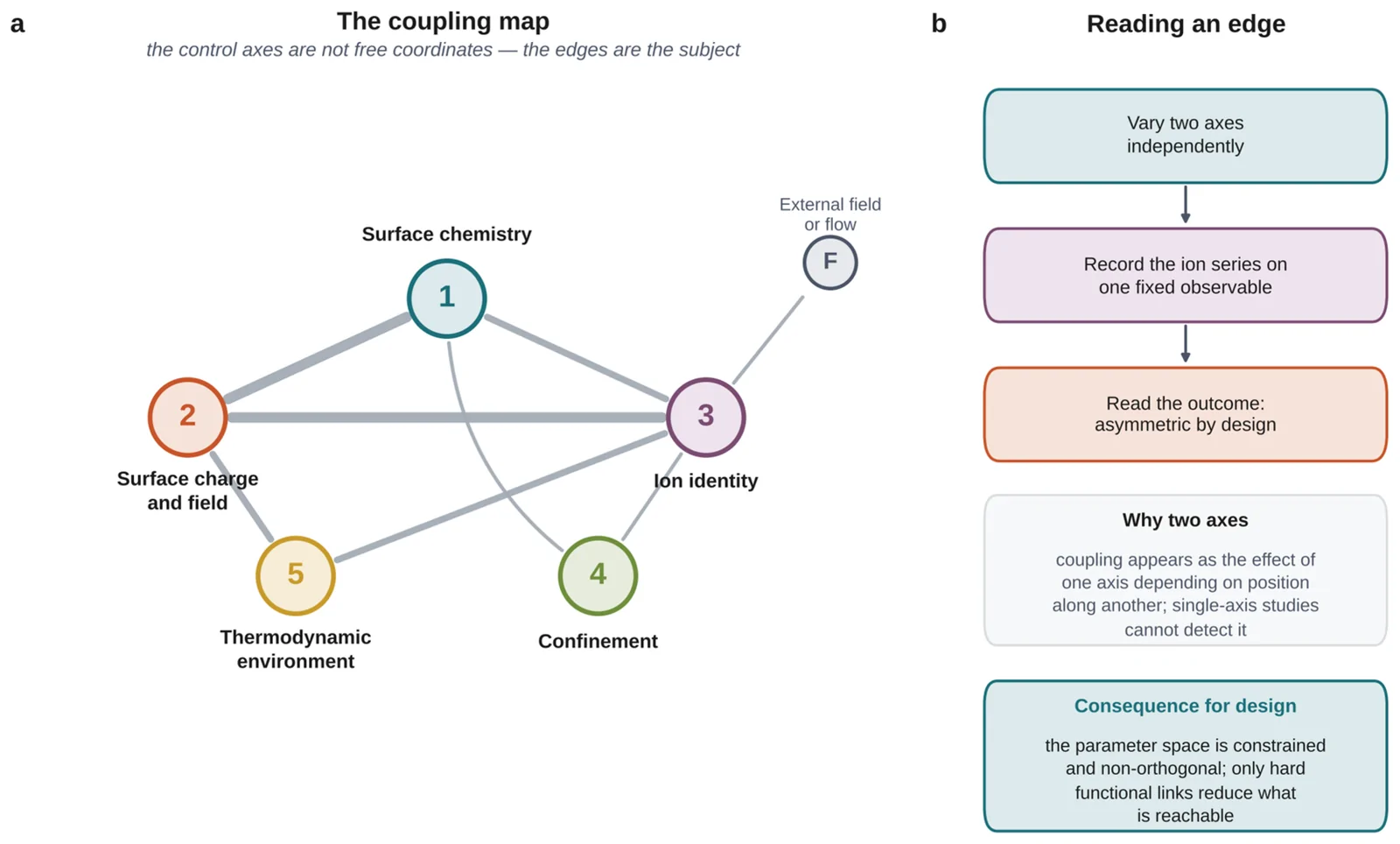
Coupling map of the five factors governing interfacial water. Edge thickness qualitatively indicates the amount of supporting evidence within the literature surveyed here and should not be interpreted as a quantitative measure of coupling strength; diagnostic criteria are indicated within the figure: (**a**) the coupling map; (**b**) reading an edge.

**Figure 3:**
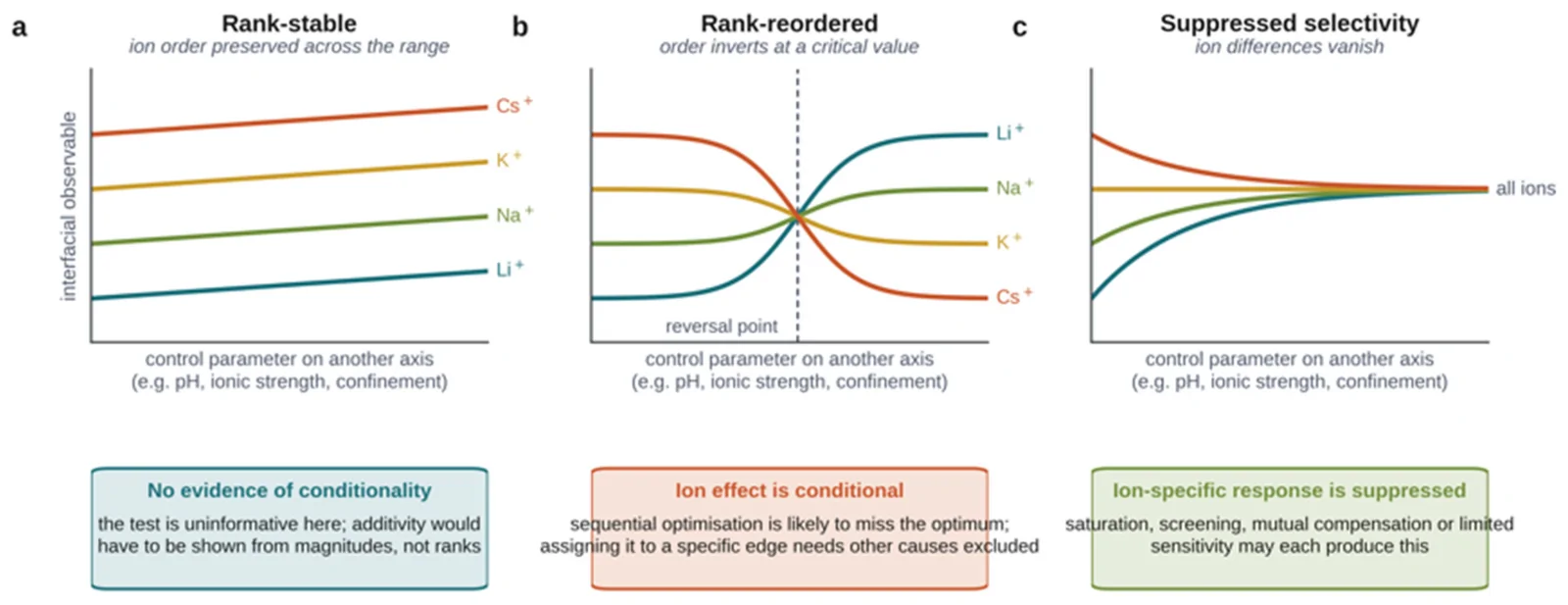
Diagnostics based on ion-series reversal. Three modes of ion-sequence behavior upon variation of the second factor and their interpretation regarding a viable optimization strategy. The curves are schematic and illustrate qualitative behavior rather than a fit to specific data: (**a**) rank-stable; (**b**) rank-reordered; (**c**) suppressed selectivity.

**Figure 4:**
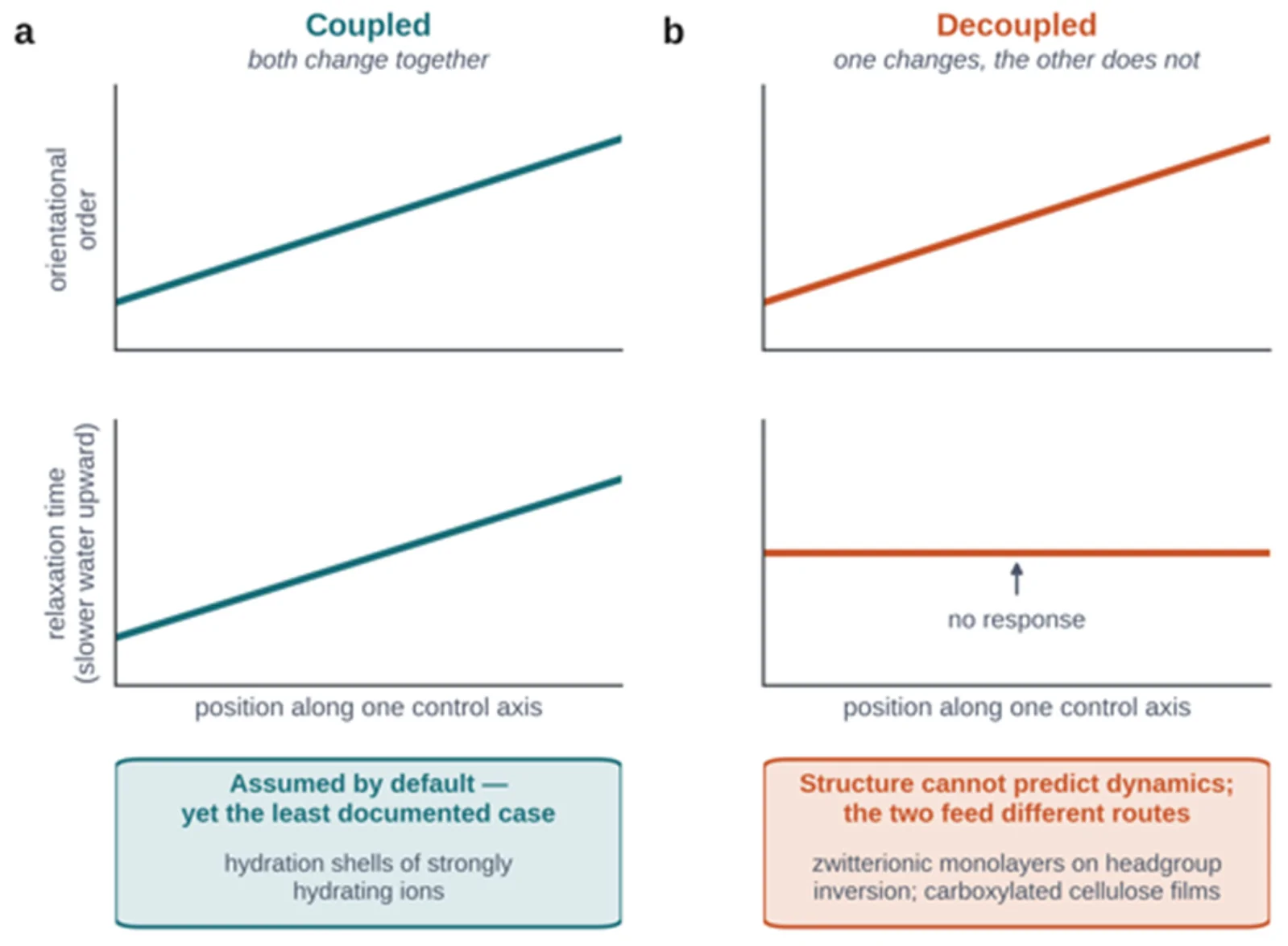
Two limiting patterns relating structural and dynamic observables during variation of a control factor. The curves are schematic and illustrate conceptual behaviors rather than their relative prevalence in the literature: (**a**) coupled; (**b**) decoupled.

**Figure 5:**
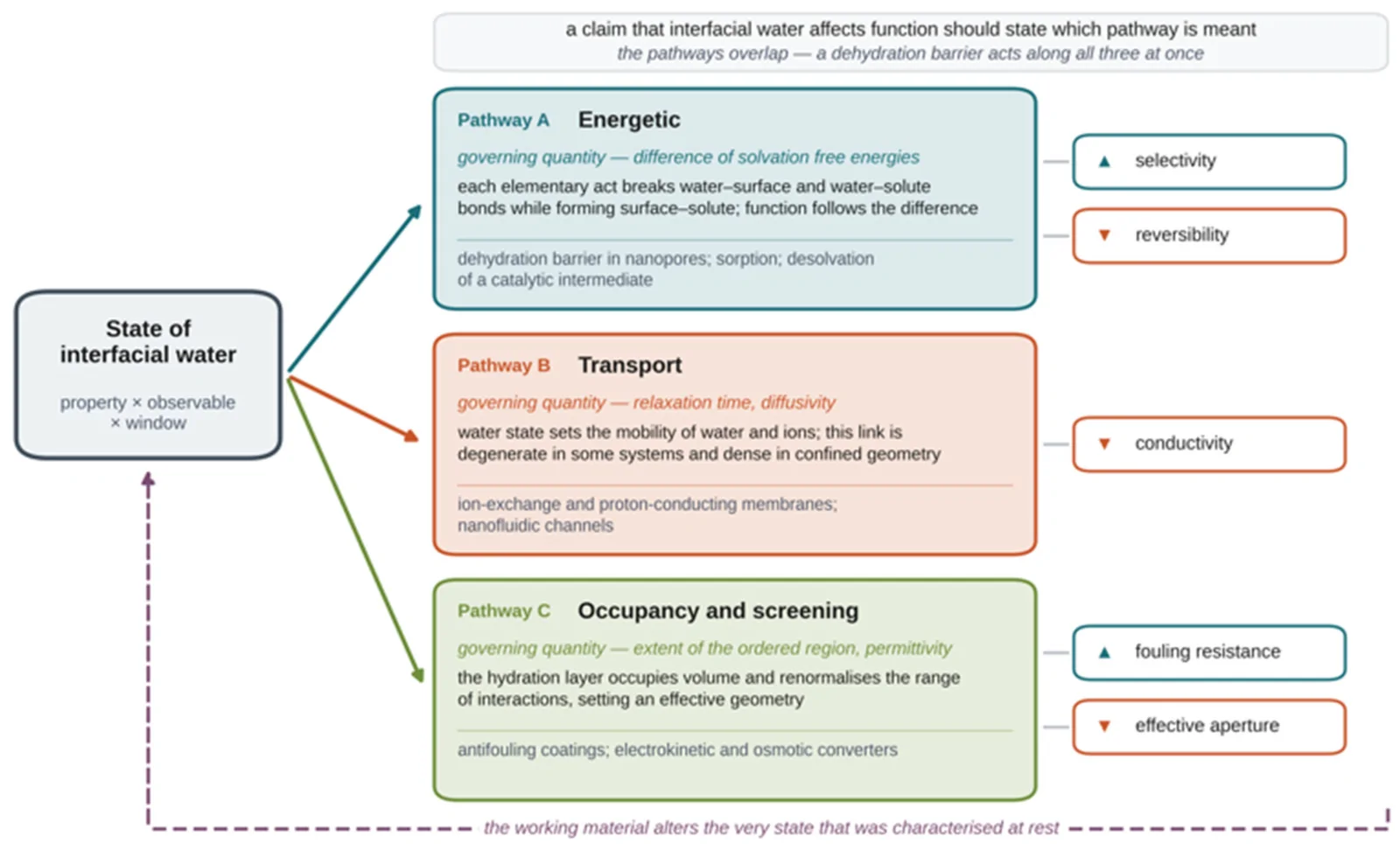
Three interpretive pathways linking interfacial-water properties to material function. The mapping is provisional and system-specific; the pathways are not mutually exclusive, and no pathway should be regarded as intrinsically dominant for an entire material class.

**Figure 6:**
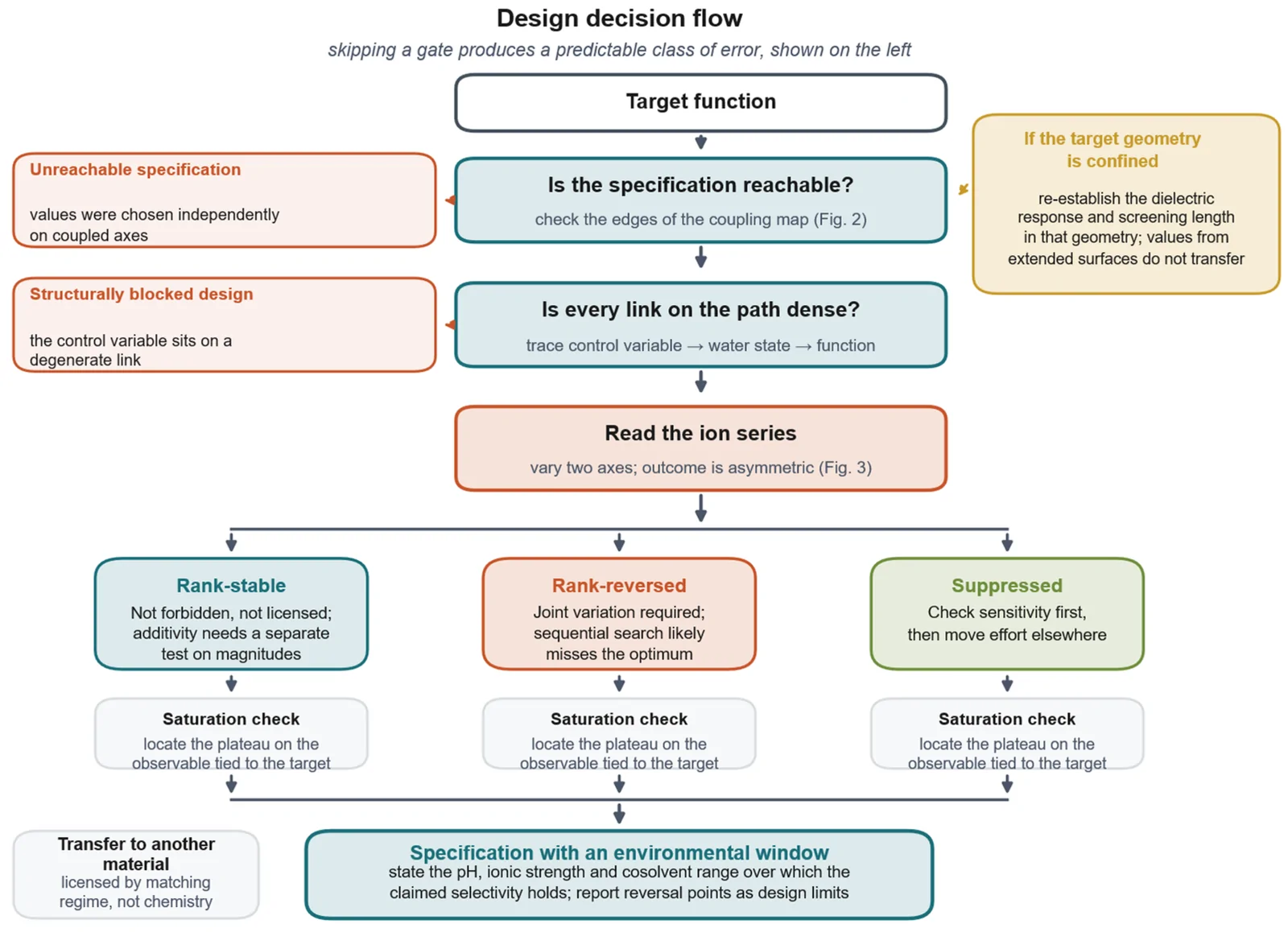
Sequence of design checks derived from the framework. Each check identifies a potential source of error or uncertainty in materials optimization. The diagram is schematic and does not imply an exhaustive or quantitatively validated decision algorithm.

**Table 1:** Commonly used terms and their operational content.

Term	Origin	Operational Content	What the Term Is Not
Interfacial water	Spatial; not tied to a specific method	Water in a region where the influence of the boundary differs from bulk behavior	Does not specify which particular property differs from the bulk
Hydration shell	Coordinational; defined relative to an ion or molecule	Water molecules in the first and subsequent coordination spheres of a solute	Does not apply to an extended surface where a coordination number is undefined
Bound water	Calorimetric and relaxation-based	A fraction that yields no heat of fusion or exchanges more slowly than a specified time	Does not imply chemical bonding; undefined without specifying a criterion and threshold
Non-freezing water	Calorimetric	A fraction that yields no thermal effect within a specified temperature range	Depends on cooling rate and instrument sensitivity; thus, not identical to bound water
Ordered water	Nonlinear-optical	Presence of a preferred orientation of the dipole moment or OH groups	Implies neither slowed dynamics nor strengthened hydrogen bonds
Structured water	No specific origin	Contains no indication of a specific property	Most frequent source of apparent discrepancies between studies
Vicinal water	Early literature; spatial	Water near a surface without specifying the extent	Usage often implies a macroscopic extent not specified by the definition
Exclusion zone	Observational	A region near certain surfaces from which colloidal particles are excluded	A description of an observation, not a statement about the state of the water

**Table 2:** Extent of the perturbed region for various properties.

Property	Measurement Method	Characteristic Extent	Boundary Criterion
Density oscillations	X-ray reflectometry; atomic force microscopy	First molecular layers	Decay of density oscillations to the bulk-average density
Charge-induced orientational ordering	Nonlinear optical spectroscopy	Determined by the Debye length and may range from nanometers to micrometers	Decay of the interfacial electric field to the level at which its orienting effect becomes comparable to thermal fluctuations
Dielectric anomaly	Capacitance measurements in nanometer-scale gaps	Nanometer scale	Deviation of the dielectric permittivity from the bulk value
Slowing of dynamics	Nuclear magnetic resonance; quasielastic neutron scattering	Typically confined to the first few layers, but system-dependent	Deviation of the diffusion coefficient or relaxation time from the corresponding bulk value
Hydration forces	Surface force apparatus	Several molecular layers	Decay of the oscillatory force component as the surfaces are separated

**Table 3:** Ion-series behavior regimes and their interpretation.

Regime	Observed Behavior	Implication for the Coupling Map
Rank-stable	The ion ranking remains unchanged when the other factors are varied	No evidence of conditionality is obtained. Additivity cannot be inferred because it is determined by effect magnitudes rather than their ranking and requires a factorial experimental design
Rank-reversed	The ion ranking changes systematically when a factor other than ion identity is varied	The ion effect is conditional on another factor or on a change in mechanism. A specific coupling edge can be assigned only after excluding changes in speciation or ion pairing, competitive adsorption, aggregation, response saturation or limited sensitivity, and rate-limiting-step changes, and after independently characterizing the proposed coupled factor
Selectivity-suppressed	Differences between ions in the given observable disappear	The response to ion identity is suppressed. Possible causes include saturation, screening, compensation between mechanisms, or the sensitivity limit of the method; therefore, attributing the effect to saturation before these possibilities are distinguished would be premature

**Table 4:** Evidence-supported connections (edges) in the factor-coupling map.

Factor Pair	Coupling Mechanism	Diagnostic Signature	Supporting Evidence
Surface chemistry and surface charge	Surface charge arises from the acid–base equilibria of the same surface groups that determine the donor–acceptor balance.	Shift in surface pKa or protonation state at constant bulk composition.	Previously established [14,15]; summarized in [5].
Surface charge and ion identity	Ion adsorption can redefine the effective density and sign of surface charge, while specific counterions alter surface protonation equilibria.	Charge inversion or counterion-dependent changes in the fraction of protonated/deprotonated surface sites.	Previously established [22,23,24,25]
Thermodynamic/chemical environment and surface charge	pH and ionic strength alter surface protonation equilibria and therefore the effective surface charge at otherwise unchanged surface chemistry.	Change in surface protonation, surface potential, or charge state as pH or ionic strength is varied.	[15,23,27]
Surface chemistry and ion identity	The identity and density of surface functional groups determine ion-specific adsorption and hydration, while specific ions can alter surface acid–base equilibria.	Change in ion adsorption or ion ranking when surface functional-group chemistry is changed under otherwise matched conditions.	[24,25,33,67]
Ion identity and thermodynamic/chemical environment	pH, ionic strength, solvent, and cosolvents alter the relative effectiveness of ions, potentially leading to reversal of ion ranking.	Reversal of the ion series upon variation of a defined environmental parameter.	[27,28,29,30]
Ion identity and confinement	Confinement modifies the anisotropic dielectric response, local ion distributions, and effective hydration environment, thereby changing ion-specific behavior.	Reduced out-of-plane dielectric response, departures from local electroneutrality, or changes/suppression of ion selectivity under confinement.	[2,52,56,78]
Surface chemistry and confinement	Functional-group identity and coverage modify confined-water structure and transport within a fixed geometry.	Dependence of ion or water transport on surface functionalization or coverage at otherwise comparable confinement.	[58,59,60,61]
External perturbation and ion identity	An applied field or flow redistributes ions and can alter their relative interfacial effectiveness.	Change in ion-specific response or ranking upon application of an external field or flow at the same interface.	[31,32]
Surface chemistry and surface charge	Surface charge arises from the acid–base equilibria of the same surface groups that determine the donor–acceptor balance	Shift in surface pKa at constant bulk pH	Previously established [14,15]; summarized in [5].
Surface charge and ion identity	Ion adsorption redefines the effective density and sign of the surface charge, while the counterion shifts surface protonation equilibria	Charge inversion and counterion-dependent fraction of deprotonated surface sites	Previously established [22,23,24,25]
Ion identity and thermodynamic environment	pH, ionic strength, solvent, and cosolvents alter the relative effectiveness of ions, potentially leading to a reversal in ion ranking	Reversal of the ion series upon crossing a characteristic value of the controlling parameter	[27,28,29,30]
Ion identity and confinement	Confinement renormalizes the dielectric response of the medium and distorts ion distributions, potentially leading to deviations from local electroneutrality	Anomalously low dielectric permittivity, deviations from electroneutrality, and suppression of ion selectivity	[2,52,56,78]
Surface chemistry and confinement	The density of functional-group coverage acts as a control parameter for transport independently of the specific group identity	Monotonic dependence of ion mobility on surface coverage at constant functional-group type	[58,59,60,61]
External field and ion identity	An applied field or flow redistributes ions and changes their relative effectiveness	Change in ion ranking upon application of the external perturbation at the same surface	[31,32]

**Table 5:** Relationship between structure and dynamics during a shift along a factor.

Case	Observed Behavior	Representative System	Implication
Coupled	Structure and dynamics change consistently as the controlling factor is varied	Hydration shells of strongly hydrated ions, where structural rearrangement is accompanied by anisotropic reorientation dynamics	Structural and dynamic observables covary under the conditions examined; using either as a proxy for the other requires direct validation in the system studied
Decoupled	One characteristic changes while the other remains essentially unchanged	Zwitterionic monolayers upon reversal of headgroup orientation; cellulose nanofibrils with varying carboxyl-group density	Structural changes do not reliably predict dynamic changes under the conditions examined; the two observables may therefore contribute differently to functional behavior

**Table 6:** Coupling type and inherent limitations of the families.

Method Family	Type of Interaction with Water	What Remains Inaccessible and Why
Symmetry-based	Breaking of centrosymmetry and nonlinear susceptibility	Ordered configurations with oppositely oriented neighboring layers, in which local symmetry is restored and the nonlinear response is suppressed
Mechanical	Force and density gradient normal to the surface	Dipole orientation, because these methods probe density rather than molecular orientation
Nuclear	Interaction with nuclear magnetic moments or neutron scattering from atomic nuclei	Spatial position relative to the interface, because the signal is collected from the entire sample volume
Electronic	Photoabsorption and scattering cross-sections	Molecular dynamics, because the signal acquisition time is substantially longer than the timescale of molecular motion
Collective polarization	Collective polarization response of the medium	Distinction between individual molecular configurations, because the measured quantity represents an ensemble-averaged response
Transmission vibrational	Absorption or inelastic-scattering cross-section of a vibrational transition	Separation of interfacial and bulk contributions when the interfacial fraction of the sample is small
Probe-based	Interaction of an introduced probe with its local environment	The unperturbed state of the system, because the probe itself may modify the local environment

**Table 7:** Transformation of the raw signal into the reported value.

Method Family	Primary Signal	Required Assumptions	Derived Quantity
Symmetry-based	Sum-frequency or second-harmonic intensity	Double-layer model for converting intensity into potential; separation of second- and third-order contributions; accounting for interference and coherence length	Surface potential; degree of orientational ordering
Mechanical	Cantilever deflection or frequency shift	Model of probe–liquid interaction and the assumption that the measured force is related to local density	Density profile normal to the surface
Nuclear, magnetic resonance	Relaxation times and line shape	Exchange model between fractions and an assumed number of distinguishable fractions	Fraction of bound water; exchange times
Nuclear, neutron scattering	Dynamic structure factor	Model of molecular motion and separation of translational and rotational contributions	Mobility coefficients
Electronic	Photoelectron spectrum or reflectivity curve	Model of electron mean free path or density-profile model used for fitting	Concentration and electron-density profiles
Vibrational	Absorption or scattering spectrum	Decomposition of the spectral band into components and assignment of these components to distinguishable states	Fractions of states with different hydrogen-bond strengths
Computational	Particle trajectories	Force field or level of theory; simulation cell size; trajectory length; boundary conditions	Any quantity accessible within the accuracy limits of the adopted model

**Table 8:** Combinations of methods resolving various types of discrepancies.

Type of Discrepancy	What Needs to Be Established	Suitable Combination
Difference in the measured observable	Measurement of two properties on the same sample	A symmetry-based method combined with a nuclear or neutron-scattering method, providing complementary information on orientation and mobility for the same sample under matched conditions
Difference in the temporal window	Overlap of the sensitivity windows	Neutron scattering combined with magnetic resonance or dielectric spectroscopy
Difference in the probed region	Independent determination of the signal-collection region	A symmetry-based method with variable reflection geometry combined with a mechanical method
Unmeasured surface state	Independent determination of the degree of protonation	An electronic method or acid–base titration combined with any method probing water
Hidden variation along a coupled factor	Control of the system’s position along all relevant factors	Any water-sensitive method combined with simultaneous independent measurements of surface state and solution composition
Difference in sample history	Standardization of sample preparation	No additional method is required; a sufficiently detailed preparation protocol is adequate
Model used to extract the quantity	Comparison of two independent extraction approaches	Nonresonant second-harmonic generation combined with resonant sum-frequency generation on the same sample

**Table 9:** Projections of the state of interfacial water recorded by major methods.

Method	Measured Quantity	Temporal Window	Spatial Region
Vibrational sum-frequency generation	Average orientational ordering of OH groups, weighted by hyperpolarizability	Steady-state/time-averaged; femtosecond–picosecond resolution only in time-resolved implementations	Region of broken centrosymmetry, supplemented by the contribution from the electric double-layer field
Second-harmonic generation	Nonresonant response converted into surface potential	Steady-state/time-averaged; ultrafast resolution only in pump–probe or time-resolved implementations	At low ionic strength, comparable to the Debye length and therefore dependent on solution composition
Atomic force microscopy and surface force apparatus	Force and density profiles normal to the surface; lateral distribution	Quasi-static	First molecular layers, with three-dimensional spatial resolution
Nuclear magnetic resonance	Relaxation times, exchange, and local nuclear environment	Nanosecond to microsecond	Entire sample volume, with differentiation by chemical shift
Quasielastic neutron scattering	Translational and rotational mobility coefficients	Picosecond to nanosecond	Entire sample volume, with discrimination according to the spatial scale of motion
Dielectric spectroscopy	Collective polarization response	Picosecond to microsecond	Entire sample volume, averaged over spatial heterogeneities
X-ray reflectometry and photoelectron spectroscopy	Electron-density profile and core-level binding energies	Quasi-static	Determined by the electron mean free path or the reflection geometry
Molecular dynamics	Any accessible quantity, within the accuracy limits of the force field	Determined by trajectory length	Determined by simulation cell size

**Table 10:** Minimum reporting information for cross-study comparability.

Item	What Should be Reported
Observable and its physical meaning	Specify which particular characteristic of the water state is being measured, rather than making a general statement about ordering
Temporal window of the method	Report the time range to which the signal is sensitive, because the fraction of bound water depends on the observation window
Probing depth and its dependence on solution composition	Indicate whether the probed volume changes with ionic strength and, if so, how this variation was taken into account
Surface state	Indicate whether the degree of protonation was measured independently or inferred from the bulk pH
Varied and controlled factors	Specify which factors were varied, which were held constant, and how the constancy of the controlled factors was verified
Model used to extract the reported quantity	Identify the model used to convert the measured signal into the reported quantity and state its underlying assumptions
Sample history	Describe the hydration, washing, and equilibration cycles performed before the measurement

## Data Availability

No new datasets were generated or analyzed in this review. All information discussed in the manuscript is derived from the published literature cited herein.

## Abbreviations

The following abbreviations are used in this manuscript:

AFM	Atomic force microscopy
ATR	Attenuated total reflection
DFT	Density functional theory
DNA	Deoxyribonucleic acid
DOI	Digital object identifier
MD	Molecular dynamics
NMR	Nuclear magnetic resonance
QENS	Quasielastic neutron scattering
SFG	Sum-frequency generation
SHG	Second-harmonic generation
